# Changes in physical functioning among men and women aged 50–79 years in Germany: an analysis of National Health Interview and Examination Surveys, 1997–1999 and 2008–2011

**DOI:** 10.1186/s12877-016-0377-0

**Published:** 2016-12-01

**Authors:** A. K. Buttery, Y. Du, M. A. Busch, J. Fuchs, B. Gaertner, H. Knopf, C. Scheidt-Nave

**Affiliations:** 1 Robert Koch Institute, Department of Epidemiology and Health Monitoring, General-Pape-Str. 64, 12101 Berlin, Germany; 2 Faculty of Life Sciences and Medicine, King’s College London, Guy’s Campus, London, SE1 1UL UK

**Keywords:** Physical functioning, Chronic disease, Multimorbidity, Disability, DEGS1, GNHIES98

## Abstract

**Background:**

This study examines changes in physical functioning among adults aged 50-79 years in Germany based on data from two German National Health Interview and Examination Surveys conducted in 1997–1999 (GNHIES98) and 2008–2011 (DEGS1).

**Methods:**

Using cross-sectional data from the two surveys (GNHIES98, *n* = 2884 and DEGS1, *n* = 3732), we examined changes in self-reported physical functioning scores (Short Form-36 physical functioning subscale (SF-36 PF)) by sex and age groups (50–64 and 65–79 years). Covariables included educational level, living alone, nine chronic diseases, polypharmacy (≥5 prescribed medicines), body mass index, sports activity, smoking and alcohol consumption. Multimorbidity was defined as ≥2 chronic diseases. Multivariable models were fitted to examine consistency of changes in physical functioning among certain subgroups and to assess changes in mean SF-36 PF scores, adjusting for changes in covariables between surveys.

**Results:**

Mean physical functioning increased among adults aged 50–79 years between surveys in unadjusted analyses, but this change was not as marked among men aged 65–79 years who experienced rising obesity (20.6 to 31.5%, *p* = 0.004) and diabetes (13.0 to 20.0%, *p* = 0.014). Prevalence of multimorbidity and polypharmacy use increased among men and women aged 65–79 years. In sex and age specific multivariable analyses, changes in physical functioning over time were consistent across subgroups. Gains in physical functioning were explained by improved education, lower body mass index and improved health-related behaviours (smoking, alcohol consumption, sports activity) in women, but less so among men.

**Conclusions:**

Physical functioning improved in Germany among adults aged 50–79 years. Improvements in the population 65–79 years were less evident among men than women, despite increases in multimorbidity prevalence among both sexes. Changes in health behaviours over time differed between sexes and help explain variations in physical functioning. Targeted health behaviour interventions are indicated from this study.

**Electronic supplementary material:**

The online version of this article (doi:10.1186/s12877-016-0377-0) contains supplementary material, which is available to authorized users.

## Background

As the worldwide phenomenon of unprecedented human longevity becomes progressively realised, how we understand, measure and improve health in later life is increasingly shaping our approaches to population monitoring and public health. Globally, investigators are shifting analytical strategies beyond crude measures of mortality and single diseases. Efforts now concentrate on measures interlinked with healthcare costs, such as healthy life expectancy that focus on the number of years a person at a given age can expect to live in good health given their age-specific mortality, morbidity and functional health status [1, 2]. Findings from the Global Burden of Disease study indicate that in many countries increases in life expectancy over the last two decades have occurred alongside a rise in the number of years spent in disability [2], an expansion of morbidity [3]. These findings emphasise the relatively minor progress made in reducing the overall effects of chronic diseases on population health compared with recent successes in increasing survival [4].

Multimorbidity, the coexistence of two or more chronic conditions [5] is now the standard for many older people in developed countries [5, 6]. Major consequences of multimorbidity include functional decline, disability, poor quality of life and high healthcare costs [7]. Changing patterns of activity limitations have been the focus of several national [8, 9] and international studies [10, 11] using large population datasets including older adults. Findings have been mixed, with activity limitations reported as generally stable over time in the Netherlands [9], particularly among those with severe disability [12] but reducing in several northern European countries [10, 13, 14]. In the US, there is evidence that mobility disability has increased over time, particularly among certain groups, such as those with obesity [15]. Recent evidence from the US has revealed widening gaps between sexes and over the last 30 years men’s active life expectancy at age 65 years has increased by 4.5 years (to more than 15 years), but only by 1.4 years for women (to more than 14 years) [16].

Germany is currently the second oldest population in the world with 27.6% of the population aged 60 years or over, surpassed only by Japan (33.1%) [17]. Chronic ischaemic heart disease (IHD) is by far the leading cause of mortality in the country, followed by stroke; and IHD is one of the most common causes of lost healthy years, particularly among men [18]. Multimorbidity is common, particularly for those aged 50 years and over with prevalence markedly increasing with age [19]. Little is known about temporal changes in physical functioning at a population level in Germany and investments to develop public health interventions and optimise health care for older people include enhanced comprehensive population health monitoring and epidemiological studies which enable surveillance over time [20, 21].

Against this background, this study investigates changes in physical functioning over time in two national health surveys of adults in Germany conducted in 1997–1999 and 2008–2011. We investigate whether changes in physical functioning were consistent within age and sex strata of the population aged 50–64 and 65–79 years. We further examine if any changes can be explained by changes in health determinants, including sociodemographic and behavioural risk factors, and changes in the prevalence of chronic disease.

## Methods

### Study design and study population

Our study draws on cross-sectional data from two national German health surveys carried out by the Robert Koch Institute as part of a continuous health monitoring programme about a decade apart [20]. The German National Health Interview and Examination Survey 1998 (GNHIES98) was conducted from October 1997 to March 1999 and the German Health Interview and Examination Survey for Adults (DEGS1) from November 2008 to December 2011. These surveys are based on nationally representative samples of the resident population in Germany aged 18–79 years. People unable to provide written consent and those with significant language barriers were excluded from participation [20].

The design and sampling procedures of the surveys have been previously reported in detail [20, 22, 23]. Both surveys used two-stage random sampling procedures. Primary sample units (PSUs) are the communities. These were randomly sampled from a complete list of German communities proportional to community size. Within PSUs, random samples of the population 18–79 years were drawn from local population registries. GNHIES98 was based on 120 PSUs and included a total of 7124 persons 18–79 years of age and had a response rate of 61%. In DEGS1, the number of PSUs was extended to 180 as surviving GNHIES98 participants were invited to participate in DEGS1 in order to establish a survey panel component. Random sampling in DEGS1 at the individual level was conducted to maintain a nationally representative sample at the population level [20]. Overall 7115 persons 18–79 years of age participated in DEGS1 and completed both the interview and examination survey parts. Response rates in DEGS1 were considerably higher among persons who also participated in GNHIES98 (response rate: 64%) compared to those newly sampled for DEGS1 (response rate: 42%).

**Fig. 1:**
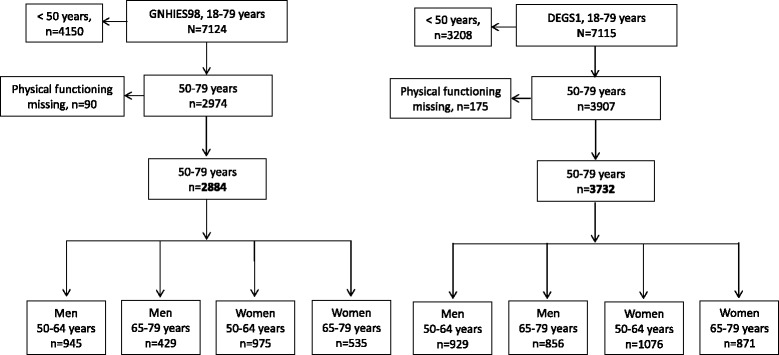
Flow diagram of study sample

Both surveys included self-administered questionnaires, standardised physician-administered computer-assisted personal interview (CAPI), physiological measurements and tests [20, 24] and robust data collection on the use of medicines in the seven days prior to examination [25]. In the present analysis, we included participants aged 50–79 years with complete interview and examination data and information on physical functioning (Fig. 1).

Participants provided written informed consent prior to interview and examination in both studies. Both studies were conducted according to the Federal and State Commissioners for Data Protection guidelines. DEGS1 was approved by the Charité-Universitätsmedizin Berlin ethics committee (No. EA2/047/08).

### Physical functioning

Validated self-administered German language versions [26] of the Medical Outcome Short Form-36 (SF-36) [27] were used in both surveys [28]. The self-reported 10-item physical functioning subscale (SF-36 PF) was the focus of this analysis. The SF-36 PF subscale is a valid marker of mobility disability in epidemiological studies of older people [29], and may be less susceptible to changes in living environments and technology over time than other measures of activities of daily living disability [30]. SF-36 PF scores range from 0 to 100 points with higher values indicating better physical functioning [31].

### Covariables

A number of covariables with known associations with physical functioning were investigated in this study. Socio-demographic variables included age, sex and household size (living alone or with others). Education level was classified as low, medium and high using the Comparative Analysis of Social Mobility in Industrial Nations (CASMIN) scale [32].

Body mass index (BMI) was calculated using the standard formula (weight (kg)/[height (m)]^2^), and categorised into <25 kg/m^2^, 25–29.9 kg/m^2^ and >30 kg/m^2^ [33]. Smoking status was classified as current, former or never smoking. Alcohol consumption was classified as risky drinking (consuming ≥10 g of alcohol per day for women and ≥20 g per day for men); moderate consumption (<10 g per day for women and <20 g per day for men) and no alcohol consumption (0 g per day) [34]. Responses about the frequency and duration of engagement in any sports activities in the previous three months [35] enabled individuals to be classified as performing ≥2 h per week; less than 2 hours per week; and no sports.

Self-reported physician-diagnosed prevalence of chronic diseases was measured in both surveys (‘Has a doctor ever told you that you have …?’). Nine diseases, with comparable data were available including: ischemic heart disease (IHD) (including myocardial infarction and angina pectoris), chronic heart failure, stroke, hypertension, hyperlipidaemia, cancer (history of any type), asthma, osteoporosis and diabetes, which additionally included participants taking diabetes medicines [36]. We classified respondents by their number of self-reported chronic conditions (0, 1, 2, 3 or more) and as having multimorbidity (2 or more chronic diseases) [5]. Data on medication use was summarised in a single measure of polypharmacy defined as the use of 5 and more prescription medicines in the 7 days prior to survey examination [25].

### Statistical analyses

IBM SPSS software (version 20.0, SPSS Inc. Chicago, IL) was used for analyses. It was necessary to take into account demographic changes in the German population between surveys. The age structure of the population changed from 1998 and numerous chronic conditions are age-related. Therefore, age-standardized sampling weights were computed for the GNHIES98 sample to the population structure as of 31 December 2010, enabling comparisons with the DEGS1 sample [36, 37]. Participants’ characteristics are summarised by survey (GNHIES98 and DEGS1). Given the potential for heterogeneity of population subgroups, we investigated sex-specific age groups 50–64 and 65–79 years, consistent with others using similar approaches [12, 16]. Prevalence estimates for each chronic condition were calculated excluding those with missing data.

A total of 509 or 7.7% of study participants had missing observations in some variables with a range of 0.2% for smoking, 0.5% for educational status, 0.8% for body mass index, to 5.0% for the number of chronic conditions. The variable ‘number of chronic conditions’ had the most missing values (*n* = 330) among all covariables ranging from 67 among men aged 50–64 years to 103 among women aged 65–79 years (Table 1). The numbers of persons with missing values are explicitly stated for each variable (Table 1). Persons with missing values were excluded from the analyses, with pairwise deletion for descriptive and listwise deletion for multivariable analyses.

**Table 1:** Characteristics of German National Health Interview and Examination Survey participants 1997–1999 (GNHIES 98) and 2008–2011(DEGS1) included in this analysis by sex and age groups

	Men						Women					
	50–64 years			65–79 years			50–64 years			65–79 years		
	GNHIES 98	DEGS1	*p*	GNHIES 98	DEGS1	*p*	GNHIES 98	DEGS1	*p*	GNHIES 98	DEGS1	*p*
	*n* = 945	*n* = 929		*n* = 429	*n* = 856		*n* = 975	*n* = 1076		*n* = 535	*n* = 871	
	%/mean	%/mean		%/mean	%/mean		%/mean	%/mean		%/mean	%/mean	
Age, years (mean ± SE)	56.4 ± .14	56.4 ± .15	.936	70.9 ± .17	71.0 ± .16	.655	56.6 ± .14	56.4 ± .15	.417	71.5 ± .17	71.3 ± .16	.374
Household size												
Living alone	6.6	13.5	**<.001**	12.5	9.6	.180	14.6	15.7	.531	41.6	32.2	**.004**
Living with others	93.4	86.5		87.5	90.4		85.4	84.3		58.4	67.8	
Educational level(CASMIN)												
Low	60.4	44.9	**<.001**	70.2	58.1	**.005**	63.4	40.9	**<.001**	82.1	69.2	**<.001**
Medium	22.1	37.9		15.5	23.1		28.0	48.0		14.6	24.2	
High	17.5	17.2		14.3	18.9		8.5	11.1		3.3	6.6	
Body mass index (BMI)kg/m^2^ (mean ± SE)	28.2 ± .15	28.2 ± .18	.773	27.8 ± .19	28.5 ± .19	**.011**	28.1 ± .22	27.4 ± .24	.275	28.6 ± .27	28.8 ± .21	.622
BMI <25^a^	18.0	20.4	.339	21.2	17.1	**.004**	31.4	36.3	.092	23.4	24.8	.336
BMI ≥ 25 to <30	54.3	50.6		58.2	51.5		39.5	35.0		42.4	38.1	
BMI ≥ 30	27.7	28.9		20.6	31.5		29.2	28.7		34.2	37.2	
Smoking status												
Current	28.7	28.0	**.003**	17.6	10.1	**<.001**	17.8	24.9	**<.001**	10.5	8.2	**<.001**
Former	37.7	45.4		58.2	51.9		15.2	33.0		10.9	20.0	
Never	33.6	26.6		24.2	37.9		67.1	42.1		78.6	71.7	
Sports activity level												
No sports (0 h/ week)	52.8	38.1	**<.001**	68.8	43.2	**<.001**	54.0	32.1	**<.001**	75.5	39.3	**<.001**
Regular sport (<2 h/week)	30.4	38.0		16.5	33.8		30.8	43.2		17.9	42.3	
Regular sport (≥2 h/week)	16.8	24.0		14.7	23.0		15.1	24.8		6.6	18.4	
Alcohol consumption												
No alcohol (0 g/day)	12.3	7.3	**<.001**	11.0	7.1	.125	26.4	16.8	**<.001**	39.4	21.3	**<.001**
Moderate drinking (<20/10 g/day, men/women)	56.8	72.7		64.9	67.9		58.8	66.4		52.8	68.8	
Risky drinking(≥20/10 g/day, men/women)	30.9	20.0		24.1	25.0		14.8	16.8		7.8	9.9	
Chronic diseases												
Ischaemic heart disease	10.3	9.3	.590	29.4	27.9	.648	5.7	3.2	**.022**	19.7	14.2	**.031**
Chronic heart failure	3.9	3.2	.515	14.3	13.5	.755	3.1	2.7	.663	16.3	11.6	.084
Stroke	1.7	2.5	.357	7.3	7.6	.882	.7	.5	.534	6.1	5.6	.714
Hypertension	33.9	47.6	**<.001**	48.2	68.0	**<.001**	33.6	41.4	**.001**	55.5	68.7	**<.001**
Dyslipidaemia	39.4	41.4	.499	33.9	51.7	**<.001**	34.9	37.4	.379	43.1	57.5	**<.001**
Diabetes mellitus	10.8	9.7	.514	13.0	20.0	**.014**	5.6	5.6	.991	16.2	18.5	.375
Cancer (any type)	3.4	4.4	.334	9.9	15.4	**.015**	7.5	10.4	**.049**	7.3	14.3	**<.001**
Asthma	4.8	5.1	.796	7.1	4.4	**.046**	6.3	7.3	.428	3.8	8.8	**.004**
Osteoporosis	2.5	2.5	.998	1.9	3.4	.209	10.3	5.7	**.003**	20.6	22.7	.457
Number of chronic diseases												
0	37.1	31.6	.197	20.3	11.4	**<.001**	35.1	31.1	.282	16.4	9.8	**.018**
1	31.6	32.7		33.2	25.3		34.1	38.4		25.2	23.1	
2	18.4	22.1		20.9	29.9		21.8	21.0		27.4	29.8	
≥3	12.9	13.6		25.6	33.4		9.0	9.6		30.9	37.3	
Multimorbidity												
≥ 2 chronic diseases	31.3	35.7	.105	46.6	63.3	**<.001**	30.8	30.5	.897	58.3	67.1	**.010**
Polypharmacy												
≥5 prescribed medications	8.2	10.7	.158	26.3	34.1	**.010**	12.4	12.6	.900	30.2	37.0	**.038**

All percentages and means were weighted and standardized to the population of 31.12.2010

*p* values refer to differences between GNHIES98 and DEGS1 (Second-order Rao-Scott chi-square tests for categorical variables and general linear models for continuous variables)

^a^Includes 20 persons with BMI <18.5 kg/m^2^

Missing values: Household size (men 50–64 years *n* = 7, men 65–79 years *n* = 3; women 50–64 years *n* = 9, women 65–79 years *n* = 13), Educational status (9, 6; 7, 8), Body mass index (7, 8; 11, 26), Smoking status (2, 1; 3, 6), Physical activity level (11, 10; 12, 10), Alcohol consumption (23, 14; 16, 20), Ischaemic Heart Disease (11, 19; 22, 28), Chronic heart failure (21, 38; 29, 41), Stroke (6, 9; 10, 8), Hypertension (13, 7; 12, 5), Hyperlipidaemia (29, 28; 21, 24), Diabetes (8, 4; 13, 7), Cancer (6, 6; 11, 5), Asthma (7, 9; 14, 10), Osteoporosis (14, 11; 22, 24), Multimorbidity/No. of chronic conditions (67, 86; 74, 103 ), Polypharmacy (6, 3; 8, 3)

Differences between participant’s characteristics in GNHIES98 and DEGS1 were examined using second-order Rao-Scott chi-square tests for categorical variables. We used the SPSS Complex Samples General Linear Model (CSGLM) procedure to measure the change in the mean physical functioning between the surveys with adjustment of covariables in multivariable models. To enable international comparability and comparisons between SF-36 versions 1 and 2 used in the surveys we calculated “norm-based scoring” whereby the SF-36 scales were first z-transformed using the average values and standard deviations of the 1998 American normative random sample [28]. Full details on subscale items, scoring procedures and handling partially missing data are provided (Additional file 1).

In order to investigate whether changes in physical functioning were consistent within age and sex strata of the population 50–64 and 65–79 years of age we fitted multiple age and sex specific models. We further examine if any change can be explained by changes in health determinants including sociodemographic and behavioural risk factors and changes in the prevalence of chronic disease. Multivariable models were restricted to participants with valid data on all model covariables. Unadjusted and fully adjusted models are presented and sensitivity analyses substituting variables that are likely to be closely associated i.e. replacing polypharmacy for multimorbidity were conducted. Interaction tests were performed to examine interactions between surveys and key covariables (educational level, BMI categories, multimorbidity) within age and sex stratified analyses.

## Results

Table 1 summarises the characteristics of the German National Health Interview and Examination Survey participants in 1997–1999 and 2008–2011, stratified by age groups and sex.

The proportion of older women (65–79 years) living alone decreased between the surveys whilst men (50–64 years) living alone increased. Educational levels and self-reported sports activity levels were higher in the more recent survey for all age and sex groups. The proportions of men aged 65–79 years with obesity rose between the survey periods. There were changes in smoking status and differing patterns occurred for men and women with improved smoking habits among older men, but the proportion of women aged 50–64 years smoking increased. Moderate drinking increased significantly in all subgroups except for men aged 65–79 years, but improvements in risky drinking occurred only in men aged 50–64 years.

Prevalence estimates between surveys changed for some chronic diseases: IHD declined among women; hypertension rose for men and women in both age groups; diabetes rose among older men; asthma rose among older women but declined among older men. Multimorbidity and polypharmacy use significantly rose among men and women aged 65–79 years.

**Fig. 2:**
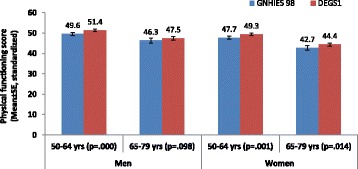
Mean SF-36 physical functioning scores among participants in the German National Health Interview and Examination Survey 1997–1999 (GNHIES 98) and 2008–2011 (DEGS1) included in this analysis by sex and age groups

Figure 2 shows physical functioning (unadjusted mean SF-36PF scores) which increased significantly between the surveys for all groups except men aged 65–79 years. In cross-sectional analyses, men had consistently higher mean physical functioning than women. Improvements in physical functioning occurred despite increasing multimorbidity.

We examined SF-36PF scores and their associations with all covariables across age groups and sex (see online Additional file 2: Table S1 for full details). Some consistent patterns for both sexes were found, including linear relationships between better physical functioning and more sports activity per week; and poorer physical functioning among those with more chronic diseases and higher use of polypharmacy. Smoking was associated with lower SF-36PF scores only among men. There was no evidence of interactions between survey period and educational level, BMI category or multimorbidity status (Additional file 2: Table S1).

Table 2 summarises the unadjusted and fully adjusted multivariable linear regression models assessing the absolute change in mean SF-36PF score between the surveys, by sex and age groups.

**Table 2:** Multivariable analysis of the absolute change in the mean SF-36 physical functioning subscale score among participants in the German National Health Interview and Examination Survey 1997–1999 (GNHIES 98) and 2008–2011 (DEGS1) included in this analysis by sex and age groups

		Men					Women				
		Change in mean	95% CI		*p*	R^2^	Change in mean	95% CI		*p*	R^2^
50–64 years	DEGS1 vs. GNHIES 98 (Unadjusted)	1.78	0.86	2.71	**<.001**	.011	1.61	0.71	2.52	**.001**	.008
	DEGS1 vs. GNHIES 98 (Adjusted^a^)	1.63	0.79	2.46	**<.001**	.142	0.36	−0.61	1.33	.466	.155
65–79 years	DEGS1 vs. GNHIES 98 (Unadjusted)	1.11	−0.21	2.44	.098	.003	1.66	0.33	2.99	**.014**	.006
	DEGS1 vs. GNHIES 98 (Adjusted^a^)	0.68	−0.79	2.15	.360	.131	0.43	−1.05	1.92	.567	.137

Weighted and standardized to the population of 31.12.2010

Change in mean: Derived from SPSS Complex Samples General Linear Model (CSGLM) with physical functioning as the dependent variable

^a^adjusted for living alone (yes vs. no), education (low, medium, high), BMI (<25, 25–30, ≥30 mg/m^2^), smoking status (current, former and never), regular sports activity (0, <2, ≥2 h/week), alcohol consumption (no, moderate and risky drinking), multimorbidity (yes, no)

Figures in bold denote statistical significance (p < .050)

Full models with all covariables are available online (Additional file 3: Table S2). For men and women rising physical functioning was partially explained by rising educational levels between survey periods (Additional file 3: Table S2). Multimorbidity appeared to contribute to changes in physical functioning particularly among older men and women (Additional file 3: Table S2 models 7 and 9). The influence of health behaviours and obesity in models had different effects for men and women (Additional file 3: Table S2, models 3–6). Behavioural health risks explained rising physical functioning among women at all ages, particularly improved sports activity levels, and despite increased smoking prevalence (Additional file 3: Table S2). There was no evidence of interactions between survey period and strata of education, BMI or multimorbidity status among men and younger women. There was some evidence of interaction between survey period and BMI for older women (see Additional file 2: Table S1 for full details). Sensitivity analyses involved substituting polypharmacy for multimorbidity and results were similar (Additional file 3: Table S2).

## Discussion

Physical functioning (SF-36PF subscale scores) improved in the German population aged 50–79 years between 1997–99 and 2008–11, but changes over time differed by sex and age groups (50–64 and 65–79 years). Increases in mean SF-36PF over time were of similar magnitude among men and women aged 50–64 years and among older women, but were less notable among older men in unadjusted models. Rising obesity and diabetes among older men found in this study may help explain this finding. Improvements in physical functioning among persons 65–79 years occurred despite increases in multimorbidity prevalence and polypharmacy use over the same period. Multivariable analyses indicated that gains in physical functioning were explained by improved education, lower BMI and improved health-related behaviours (smoking, alcohol consumption, sports activity) in women, but less so among men.

### Increasing physical functioning

Valid comparisons with other large-scale studies examining changes in physical functioning over time is challenging due to the range of activity limitations instruments available, variations in the age of study participants and different analytical techniques. Our findings are consistent with a Dutch study of older non-institutionalised population aged 55–84 years, conducted from 1990 to 2008, reporting slight decreases in the prevalence of activity limitations for SF-36 items over time, despite rising chronic diseases prevalence [38]. One potential explanation for these findings is that diseases have become less disabling and have less impact on physical functioning [38]. In a recent study in the US, investigating activity limitations across five national datasets, the percentage of the population 65–84 years with one or more activity limitation was found to be stable since 2000 and declines in the 1980s and 1990s have paused [39]. However, for those less than 65 years our finding of improved physical functioning over time differs from observations in the US [39] and in France [40]. In these previous studies, an expansion of certain disability dimensions for those in mid-life (aged 50–65 years), has raised concerns about rising prevalence of functioning problems and disability for this age group but different measurement instruments may explain why our findings are more consistent with the Dutch study [38].

### Sex differences

We observed better physical functioning among men than women in cross-sectional analyses, consistent with a previous cross-sectional study of adults aged 55–89 years in Denmark and Russia using the SF-36 PF subscale [41]. Men have been reported to have lower prevalence of functional limitations and disability consistently across many countries including continental Europe, the UK and the US [10, 42]. The male–female health survival paradox [43], which describes how men live shorter but healthier lives than women, may help explain this finding which has occurred in the context of rising life expectancy more so for men than for women in Germany over this period [44].

We found significant rises in polypharmacy use among older men and women, consistent with population studies in Sweden [45] and the US [46]. Earlier diagnoses of conditions that influence cardiovascular health such as hypertension, hyperlipidaemia and diabetes and improved access and use of cardioprotective medicines [25] may explain rising polypharmacy. Declining use of menopausal hormone therapy, a phenomenon also described in US populations over this period [47], may have influenced polypharmacy results among women.

### Explanatory variables

Unsurprisingly, lower education, increasing BMI, lower levels of sports activity, and a more adverse health status (increasing number of chronic health conditions and polypharmacy) were consistently associated with lower physical functioning in all age and sex groups and in both surveys. In accordance with previous results [15], changes in physical functioning over time were less favourable among persons with obesity compared to non-obese persons in the present study, albeit results of interaction tests were not significant. There was no evidence for a differential change in physical functioning over time according to educational level or multimorbidity status. Modifiable lifestyle risk factors have been associated with poorer physical functioning (SF-36) among adults aged 59 to 73 in England, with the more risk factors present, the worse the physical functioning [48]. However, in our study the contributions of these variables to explain changes in physical functioning were inconsistent across age and sex strata. Education improved over time in all subgroups, and helped to explain improved physical functioning particularly among older women in multivariable models. Among younger women, behavioural factors including improved BMI and sports activity levels appeared to play a larger role in explaining changes in physical functioning. Smoking has specifically been associated with poorer physical performance among women [49] and our concerning finding of rising smoking prevalence among women aged 50–64 years suggests that smoking cessation strategies need to target these women who lag behind men in the tobacco epidemic [50].

### Strengths and limitations

Strengths of our study include using nationally representative data and standardised valid data collection tools to assess changes in physical functioning, chronic health conditions, and various health determinants. However, people with severe disease and impairments, including cognitive impairment, are likely to be under-represented due to the survey age range (18–79 years) and the need to travel to survey sites for examinations. Therefore, functional limitation prevalence estimates may be underestimated.

Our study was limited by comparable data availability on chronic diseases in both surveys. Important conditions such as chronic obstructive pulmonary disease (COPD), musculoskeletal disorders and mental illness that may impact on physical functioning are missing from this analysis. Although, older people self-report certain diseases (such as cardiovascular events and diabetes) fairly accurately [51], conditions such as heart failure may be less reliably reported [52] and under and over reporting may have occurred. We defined diseases in terms of their presence but did not measure disease severity.

### Implications

Retaining a high level of functioning, in addition to living until old age without major morbidity and disability, is a central concept of healthy ageing [53]. Monitoring population health and understanding different dimensions of physical functioning and disabilities and their relationships with chronic disease is increasingly important for societies with ageing populations and rising multimorbidity. Established multifactorial inventions that improve physical functioning and maintain independence for older people [54] require increased implementation and our results suggest further attention is needed on developing and delivering effective interventions targeting health risk factors such as smoking, BMI, and diabetes while considering sex and birth cohort specific changes in health risks profiles.

Measuring functional status objectively with performance tests may contribute to our understanding of the relationships between diseases, objective functioning, and self-rated disability [38]. Refined comprehensive comparable health indicators including both physical and cognitive functioning are essential to fully understand and improve health trends among ageing societies and to investigate compression or expansion of morbidity and disability [3, 55, 56].

## Conclusion

This nationally representative study demonstrates that physical functioning improved over the first decade of the 21^st^ century among adults 50–79 years in Germany. Improvements in the population 65–79 years were less evident among men than women, despite increases in multimorbidity prevalence and polypharmacy use among both sexes. These changes occurred alongside improvements in some, but not all, modifiable health determinants with considerable differences across age group and sex groups. Higher education, lower BMI, and healthier behaviours, in particular higher sports activity were consistently associated with better physical functioning. Changes in physical functioning were no less favourable among subgroups with multimorbidity or lower education, and those with obesity. Changes in health risk behaviours and their contribution to change in physical functioning varied by age and sex. Targeted health behaviour interventions are indicated from this study, along with continued and comprehensive surveillance of morbidity and functioning.

## Additional files

Additional file 1: Physical Functioning measurement. (DOCX 23 kb)
Additional file 2: Table S1. Mean SF-36 physical functioning scores of German National Health Interview and Examination Survey participants 1997–1999 (GNHIES98) and 2008–2011 (DEGS1) included in this analysis by sex, age-groups, survey and covariables. (DOCX 33 kb)
Additional file 3: Table S2. Multivariable analysis of the absolute change in the mean SF-36 physical functioning subscale score among participants in the German National Health Interview and Examination Survey 1997–1999 (GNHIES98) and 2008–2011 (DEGS1) included in this analysis by sex and age groups including results for all covariables. (DOCX 70 kb)

## Abbreviations

BMI: Body mass index
CAPI: Computer-assisted personal interview
CASMIN: Comparative Analysis of Social Mobility in Industrial Nations scale
CSGLM: SPSS Complex Samples General Linear Model procedure
DEGS1: German Health Interview and Examination Survey for Adults
GNHIES98: German National Health Interview and Examination Survey 1998
IHD: Ischemic heart disease
SF-36 PF: Medical Outcome Short Form-36 physical functioning subscale
