# Supplementary material for: Changes in physical functioning among men and women aged 50–79 years in Germany: an analysis of National Health Interview and Examination Surveys, 1997–1999 and 2008–2011
Source: BMC Geriatr. 2016 Dec 1;16:205. doi: 10.1186/s12877-016-0377-0 (PMC5134286; doi:10.1186/s12877-016-0377-0)
Supplement: Additional file 1: — Physical Functioning measurement. (DOCX 23 kb) [file 12877_2016_377_MOESM1_ESM.docx]

**Additional file 1**

**Physical Functioning measurement**

The Medical Outcome Short Form-36 (SF-36) physical functioning subscale (SF-36 PF) [[27]](#_ENREF_27) examines perceived levels of limitations and includes 10 items: vigorous activities (e.g. running and strenuous sports); moderate activities (e.g. pushing a vacuum cleaner, playing golf); lifting or carrying groceries; climbing several flights of stairs; climbing one flight of stairs; bending, kneeling or stooping; walking more than one kilometre; walking several hundred metres; walking one hundred metres; and bathing or dressing [31]. Responses are rated on a 3-point Likert scale and options include: yes, limited a lot; yes, limited a little; and no, not limited at all. There was a change in the SF-36 versions used between the surveys (SF-36 version 1 in GNHIES98 and SF-36 version 2 in DEGS1). The SF-36 PF subscale was largely unchanged between versions. However, two items on the subscale (walking several hundred metres; walking one hundred metres) had minimal changes to phrasing. Established scoring procedures were used (each item was scored as 0=limited a lot, 50=limited and 100=not limited) and scores were summed and divided by the number of items in the SF-36 PF (n=10) [31]. Therefore, SF-36 PF scores ranged from 0 to 100 points with higher values indicating greater physical functioning. To calculate SF-36 PF data we used published algorithms and imputation methods for missing items [31]. These published methods include replacing missing values with the average values of the other questions of the scale. This occurred in n=50 (or 1.7%) cases in GNHIES98 and n=337 (or 9.0%) cases in DEGS1. However, if more than half of the questions were not answered then responses for the SF-36PF subscale were classified as missing (see Figure 1).
