# Supplementary material for: Changes in physical functioning among men and women aged 50–79 years in Germany: an analysis of National Health Interview and Examination Surveys, 1997–1999 and 2008–2011
Source: BMC Geriatr. 2016 Dec 1;16:205. doi: 10.1186/s12877-016-0377-0 (PMC5134286; doi:10.1186/s12877-016-0377-0)
Supplement: Additional file 2: Table S1. — Mean SF-36 physical functioning scores of German National Health Interview and Examination Survey participants 1997–1999 (GNHIES98) and 2008–2011 (DEGS1) included in this analysis by sex, age-groups, survey and covariables. (DOCX 33 kb) [file 12877_2016_377_MOESM2_ESM.docx]

**Additional file 2**

**Table S1** Mean SF-36 physical functioning scores of German National Health Interview and Examination Survey participants 1997-1999 (GNHIES 98) and 2008-2011(DEGS1) included in this analysis by sex, age-groups, survey and covariables

|  | **Men** | | | | **Women** | | | |
| --- | --- | --- | --- | --- | --- | --- | --- | --- |
|  | **50-64 yrs** | | **65-79 yrs** | | **50-64 yrs** | | **65-79 yrs** | |
|  | Mean SF-36PF score | | Mean SF-36PF score | | Mean SF-36PF score | | Mean SF-36PF score | |
|  | GNHIES98 | DEGS1 | GNHIES98 | DEGS1 | GNHIES98 | DEGS1 | GNHIES98 | DEGS1 |
| Living alone |  |  |  |  |  |  |  |  |
| Yes | 49.96 | 49.38 | 46.08 | 46.84 | 46.58 | 48.81 | 43.44 | 43.35 |
| No | 49.53 | 51.66 | 46.42 | 47.55 | 47.94 | 49.44 | 42.30 | 44.89 |
| p value^a^ | 0.746 | 0.034 | 0.837 | 0.585 | 0.210 | 0.540 | 0.323 | 0.166 |
| Education level |  |  |  |  |  |  |  |  |
| Low | 48.06 | 50.17 | 46.27 | 46.48 | 46.76 | 47.91 | 41.96 | 43.40 |
| Medium | 51.35 | 51.90 | 45.64 | 48.17 | 49.41 | 50.16 | 46.49 | 45.70 |
| High | 52.41 | 53.26 | 47.65 | 49.87 | 50.59 | 50.81 | 44.52 | 50.07 |
| p value | <0.001 | <0.001 | 0.489 | 0.005 | <0.001 | 0.003 | 0.005 | <0.001 |
| BMI, kg/m^2^ |  |  |  |  |  |  |  |  |
| <25^b^ | 50.00 | 53.07 | 48.01 | 49.67 | 50.15 | 52.05 | 44.60 | 48.62 |
| 25-30 | 49.98 | 52.13 | 46.95 | 48.76 | 48.32 | 49.81 | 43.69 | 45.02 |
| ≥30 | 48.42 | 48.81 | 44.51 | 44.14 | 44.29 | 45.55 | 40.81 | 41.22 |
| p value | 0.111 | <0.001 | 0.095 | <0.001 | <0.001 | <0.001 | 0.019 | <0.001 |
| Smoking status |  |  |  |  |  |  |  |  |
| Smoker | 48.54 | 50.44 | 46.42 | 43.92 | 48.13 | 49.06 | 44.30 | 42.82 |
| Ex-smoker | 49.06 | 51.21 | 45.98 | 47.30 | 47.39 | 49.97 | 42.72 | 43.37 |
| Nonsmoker | 51.02 | 52.49 | 47.18 | 48.69 | 47.77 | 48.98 | 42.54 | 44.81 |
| p value | 0.006 | 0.030 | 0.663 | 0.014 | 0.871 | 0.368 | 0.693 | 0.333 |
| Sports activity level |  |  |  |  |  |  |  |  |
| No sports (0 hrs/ week) | 48.12 | 50.09 | 45.13 | 44.91 | 46.49 | 46.26 | 41.45 | 41.57 |
| Regular sports  (<2hrs/week) | 50.65 | 51.52 | 48.59 | 48.80 | 48.81 | 49.71 | 46.54 | 45.11 |
| Regular sports  (≥ 2 hrs/week) | 52.11 | 53.09 | 49.83 | 50.83 | 50.39 | 52.42 | 46.53 | 48.09 |
| P value | <0.001 | 0.001 | 0.002 | <0.001 | <0.001 | <0.001 | <0.001 | <0.001 |
| Alcohol consumption |  |  |  |  |  |  |  |  |
| No alcohol (0 g/day) | 45.13 | 46.66 | 42.02 | 43.71 | 45.38 | 45.22 | 41.56 | 41.90 |
| Moderate drinking  <20/10 g/day, men/women | 50.24 | 51.84 | 47.47 | 47.82 | 48.63 | 49.74 | 43.28 | 44.92 |
| Risky drinking  ≥20/10 g/day, men/women | 50.16 | 51.93 | 45.54 | 47.77 | 48.85 | 51.69 | 44.78 | 46.43 |
| p value | 0.001 | 0.002 | 0.046 | 0.100 | 0.005 | <0.001 | 0.155 | 0.008 |
| Number of chronic diseases |  |  |  |  |  |  |  |  |
| 0 | 52.02 | 53.28 | 51.13 | 50.50 | 49.94 | 51.04 | 46.50 | 49.61 |
| 1 | 49.22 | 53.00 | 47.68 | 50.38 | 48.45 | 50.21 | 45.24 | 47.83 |
| 2 | 49.51 | 50.57 | 46.56 | 47.92 | 45.60 | 47.57 | 42.54 | 44.41 |
| ≥3 | 43.41 | 46.34 | 40.71 | 44.26 | 41.85 | 45.41 | 38.67 | 41.88 |
| p value | <0.001 | <0.001 | <0.001 | <0.001 | <0.001 | <0.001 | <0.001 | <0.001 |
| Multimorbidity |  |  |  |  |  |  |  |  |
| Yes | 47.00 | 48.96 | 43.34 | 45.99 | 44.51 | 46.89 | 40.49 | 43.00 |
| No | 50.72 | 53.14 | 48.99 | 50.42 | 49.21 | 50.58 | 45.74 | 48.36 |
| p value | <0.001 | <0.001 | <0.001 | <0.001 | <0.001 | <0.001 | <0.001 | <0.001 |
| Polypharmacy |  |  |  |  |  |  |  |  |
| Yes | 44.02 | 43.55 | 40.79 | 43.51 | 41.53 | 42.56 | 37.15 | 39.60 |
| No | 50.06 | 52.26 | 48.34 | 49.54 | 48.64 | 50.30 | 45.07 | 47.18 |
| p value | <0.001 | <0.001 | <0.001 | <0.001 | <0.001 | <0.001 | <0.001 | <0.001 |

Weighted and standardized to the population of 31.12.2010.

^a^ p values refer to differences within the survey of GNHIES98 and DEGS1, examined using general linear models.

^b^ Includes 20 persons with BMI <18.5 kg/m².

Results of tests for interaction between survey period and selected covariables within strata of age and sex (men 50-64 yrs; men 65-79 yrs; women 50-64 yrs; women 65-79 yrs):

Education: p=0.275; p=0.281; p=0.795; p=0.135

BMI: p=0.106; p=0.382; p=0.819; P=0.096

Multimorbidity: p=0.656; p=0.396; p=0.362; p=0.933
