# Supplementary material for: Changes in physical functioning among men and women aged 50–79 years in Germany: an analysis of National Health Interview and Examination Surveys, 1997–1999 and 2008–2011
Source: BMC Geriatr. 2016 Dec 1;16:205. doi: 10.1186/s12877-016-0377-0 (PMC5134286; doi:10.1186/s12877-016-0377-0)
Supplement: Additional file 3: Table S2. — Multivariable analysis of the absolute change in the mean SF-36 physical functioning subscale score among participants in the German National Health Interview and Examination Survey 1997–1999 (GNHIES98) and 2008–2011 (DEGS1) included in this analysis by sex and age groups including results for all covariables. (DOCX 70 kb) [file 12877_2016_377_MOESM3_ESM.docx]

**Additional file 3**

**Table S2** Multivariable analysis of the absolute change in the mean SF-36 physical functioning subscale score among participants in the German National Health Interview and Examination Survey 1997-1999 (GNHIES98) and 2008-2011 (DEGS1) included in this analysis by sex and age groups including results for all covariables.

|  |  | **Men** | | | | | | | | | | **Women** | | | | | | | | | | | |
| --- | --- | --- | --- | --- | --- | --- | --- | --- | --- | --- | --- | --- | --- | --- | --- | --- | --- | --- | --- | --- | --- | --- | --- |
|  |  | **50-64 years** | | | | | **65-69 years** | | | | | **50-64 years** | | | | | **65-79 years** | | | | | | |
|  | Variables in model | Change | 95%CI | | p | R^2^ | Change | 95% CI | | p | R^2^ | Change | 95% CI | | p | R^2^ | Change | 95%CI | | p | | R^2^ | |
| **Model 0** | **DEGS1 vs. GNHIES98** | **1.78** | **0.86** | **2.71** | **<0.001** | **0.011** | **1.11** | **-0.21** | **2.44** | **0.098** | **0.003** | **1.61** | **0.71** | **2.52** | **0.001** | **0.008** | **1.66** | **0.33** | **2.99** | | **0.014** | | **0.006** |
|  |  |  |  |  |  |  |  |  |  |  |  |  |  |  |  |  |  |  |  | |  | |  |
| **Model 1** | **DEGS1 vs. GNHIES98** | **1.88** | **0.95** | **2.82** | **<0.001** | **0.013** | **1.09** | **-0.22** | **2.41** | **0.103** | **0.003** | **1.61** | **0.71** | **2.52** | **0.001** | **0.009** | **1.61** | **0.26** | **2.96** | | **0.020** | | **0.006** |
|  | Living with others vs. living alone | 1.35 | -0.37 | 3.06 | 0.124 |  | 0.50 | -1.69 | 2.68 | 0.656 |  | 0.99 | -0.49 | 2.47 | 0.190 |  | 0.11 | -1.51 | 1.72 | | 0.898 | |  |
|  |  |  |  |  |  |  |  |  |  |  |  |  |  |  |  |  |  |  |  | |  | |  |
| **Model 2** | **DEGS1 vs. GNHIES98** | **1.43** | **0.53** | **2.33** | **0.002** | **0.043** | **0.98** | **-0.34** | **2.29** | **0.144** | **0.010** | **0.91** | **0.01** | **1.80** | **0.047** | **0.027** | **1.21** | **-0.17** | **2.58** | | **0.085** | | **0.028** |
|  | Middle vs. primary education level | 2.44 | 1.46 | 3.42 | <0.001 |  | 0.61 | -1.26 | 2.47 | 0.523 |  | 2.46 | 1.28 | 3.64 | <0.001 |  | 3.19 | 1.66 | 4.73 | | <0.001 | |  |
|  | High vs. primary education level | 3.77 | 2.75 | 4.78 | <0.001 |  | 2.40 | 0.71 | 4.08 | 0.006 |  | 3.35 | 1.98 | 4.71 | <0.001 |  | 5.31 | 3.14 | 7.48 | | <0.001 | |  |
|  |  |  |  |  |  |  |  |  |  |  |  |  |  |  |  |  |  |  |  | |  | |  |
| **Model 3** | **DEGS1 vs. GNHIES98** | **1.83** | **0.89** | **2.77** | **<0.001** | **0.031** | **1.23** | **-0.05** | **2.51** | **0.060** | **0.032** | **1.56** | **0.71** | **2.41** | **<0.001** | **0.080** | **1.65** | **0.33** | **2.96** | | **0.014** | | **0.048** |
|  | BMI<25 vs. ≥30 | 2.97 | 1.58 | 4.37 | <0.001 |  | 4.63 | 2.38 | 6.88 | <0.001 |  | 6.19 | 4.98 | 7.40 | <0.001 |  | 5.62 | 3.91 | 7.33 | | <0.001 | |  |
|  | BMI 25-30 vs. ≥30 | 2.45 | 1.36 | 3.53 | <0.001 |  | 3.65 | 2.00 | 5.29 | <0.001 |  | 4.15 | 2.82 | 5.48 | <0.001 |  | 3.37 | 1.88 | 4.86 | | <0.001 | |  |
|  |  |  |  |  |  |  |  |  |  |  |  |  |  |  |  |  |  |  |  | |  | |  |
| **Model 4** | **DEGS1 vs. GNHIES98** | **1.88** | **0.95** | **2.80** | **<0.001** | **0.022** | **0.87** | **-0.44** | **2.18** | **0.191** | **0.009** | **1.45** | **0.48** | **2.43** | **0.004** | **0.008** | **1.69** | **0.34** | **3.03** | | **0.014** | | **0.006** |
|  | Ex-smoker vs. smoker | 0.66 | -0.63 | 1.96 | 0.313 |  | 0.94 | -1.41 | 3.29 | 0.430 |  | 0.33 | -1.16 | 1.82 | 0.663 |  | -0.88 | -3.71 | 1.94 | | 0.538 | |  |
|  | Non-smoker vs. smoker | 2.29 | 1.08 | 3.50 | <0.001 |  | 2.31 | -0.13 | 4.76 | 0.064 |  | -0.16 | -1.45 | 1.14 | 0.813 |  | -0.13 | -2.85 | 2.59 | | 0.926 | |  |
|  |  |  |  |  |  |  |  |  |  |  |  |  |  |  |  |  |  |  |  | |  | |  |
| **Model 5** | **DEGS1 vs. GNHIES98** | **1.39** | **0.49** | **2.30** | **0.003** | **0.040** | **0.12** | **-1.21** | **1.45** | **0.861** | **0.049** | **0.64** | **-0.26** | **1.55** | **0.162** | **0.049** | **-0.17** | **-1.56** | **1.21** | | **0.805** | | **0.049** |
|  | Sports <2 hrs/wk vs. no sports | 2.00 | 0.85 | 3.15 | 0.001 |  | 3.64 | 2.01 | 5.27 | <0.001 |  | 2.79 | 1.80 | 3.78 | <0.001 |  | 4.13 | 2.63 | 5.63 | | <0.001 | |  |
|  | Sports ≥ 2 hrs/wk vs. no sports | 3.50 | 2.32 | 4.68 | <0.001 |  | 5.35 | 3.70 | 6.99 | <0.001 |  | 5.08 | 3.81 | 6.35 | <0.001 |  | 6.24 | 4.63 | 7.85 | | <0.001 | |  |
|  |  |  |  |  |  |  |  |  |  |  |  |  |  |  |  |  |  |  |  | |  | |  |
| **Model 6** | **DEGS1 vs. GNHIES98** | **1.63** | **0.75** | **2.52** | **<0.001** | **0.045** | **0.93** | **-0.37** | **2.23** | **0.160** | **0.022** | **1.13** | **0.22** | **2.04** | **0.016** | **0.039** | **1.27** | **-0.05** | **2.59** | | **0.058** | | **0.018** |
|  | Moderate drinking vs. no alcohol | 5.12 | 3.15 | 7.10 | <0.001 |  | 4.88 | 1.89 | 7.87 | 0.002 |  | 3.74 | 2.11 | 5.37 | <0.001 |  | 2.23 | 0.51 | 3.96 | | 0.012 | |  |
|  | Risky drinking vs. no alcohol | 5.11 | 3.02 | 7.21 | <0.001 |  | 3.87 | 0.60 | 7.14 | 0.021 |  | 4.88 | 2.94 | 6.82 | <0.001 |  | 3.74 | 1.44 | 6.04 | | 0.002 | |  |
|  |  |  |  |  |  |  |  |  |  |  |  |  |  |  |  |  |  |  |  | |  | |  |
| **Model 7** | **DEGS1 vs. GNHIES98** | **2.25** | **1.36** | **3.15** | **<0.001** | **0.065** | **2.11** | **0.76** | **3.46** | **0.002** | **0.064** | **1.69** | **0.76** | **2.61** | **<0.001** | **0.052** | **2.55** | **1.17** | **3.94** | | **<0.001** | | **0.065** |
|  | Multimorbidity (no vs. yes) | 3.95 | 2.92 | 4.99 | <0.001 |  | 5.11 | 3.35 | 6.87 | <0.001 |  | 4.22 | 3.11 | 5.32 | <0.001 |  | 5.30 | 3.87 | 6.72 | | <0.001 | |  |
|  |  |  |  |  |  |  |  |  |  |  |  |  |  |  |  |  |  |  |  | |  | |  |
| **Model 8** | **DEGS1 vs. GNHIES98** | **1.95** | **1.06** | **2.84** | **<0.001** | **0.077** | **1.65** | **0.37** | **2.94** | **0.012** | **0.094** | **1.58** | **0.67** | **2.49** | **0.001** | **0.077** | **2.23** | **0.95** | **3.51** | | **0.001** | | **0.119** |
|  | Polypharmacy (no vs. yes) | 7.53 | 5.58 | 9.48 | <0.001 |  | 6.76 | 5.18 | 8.34 | <0.001 |  | 7.42 | 5.84 | 9.01 | <0.001 |  | 7.74 | 6.31 | 9.18 | | <0.001 | |  |
|  |  |  |  |  |  |  |  |  |  |  |  |  |  |  |  |  |  |  |  | |  | |  |
| **Model 9** | **DEGS1 vs. GNHIES98** | **1.63** | **0.79** | **2.46** | **<0.001** | **0.142** | **0.68** | **-0.79** | **2.15** | **0.360** | **0.131** | **0.36** | **-0.61** | **1.33** | **0.466** | **0.155** | **0.43** | **-1.05** | **1.92** | | **0.567** | | **0.137** |
|  | Living with others vs.living alone | 0.66 | -0.90 | 2.22 | 0.406 |  | 0.01 | -2.17 | 2.18 | 0.995 |  | 0.63 | -0.71 | 1.97 | 0.353 |  | 0.85 | -0.73 | 2.43 | | 0.288 | |  |
|  | Middle vs. primary education level | 1.91 | 0.90 | 2.91 | <0.001 |  | 0.03 | -1.67 | 1.72 | 0.976 |  | 1.69 | 0.68 | 2.71 | 0.001 |  | 2.43 | 0.95 | 3.90 | | 0.001 | |  |
|  | High vs. primary education level | 2.03 | 1.03 | 3.02 | <0.001 |  | 1.02 | -0.54 | 2.57 | 0.198 |  | 0.78 | -0.58 | 2.15 | 0.259 |  | 3.09 | 1.27 | 4.90 | | 0.001 | |  |
|  | BMI<25 vs. ≥30 | 1.62 | 0.31 | 2.93 | 0.016 |  | 2.33 | 0.04 | 4.62 | 0.047 |  | 4.44 | 3.15 | 5.73 | <0.001 |  | 3.75 | 1.97 | 5.53 | | <0.001 | |  |
|  | BMI 25-30 vs. ≥30 | 1.73 | 0.69 | 2.77 | 0.001 |  | 1.70 | -0.15 | 3.56 | 0.072 |  | 3.07 | 1.72 | 4.42 | <0.001 |  | 2.26 | 0.77 | 3.74 | | 0.003 | |  |
|  | Ex-smoker vs. smoker | 0.58 | -0.61 | 1.76 | 0.338 |  | -0.07 | -2.11 | 1.96 | 0.943 |  | -0.20 | -1.64 | 1.23 | 0.780 |  | 0.55 | -2.27 | 3.38 | | 0.700 | |  |
|  | Non-smoker vs. smoker | 1.55 | 0.42 | 2.68 | 0.008 |  | 1.37 | -0.73 | 3.47 | 0.199 |  | 0.01 | -1.18 | 1.21 | 0.983 |  | 1.07 | -1.73 | 3.87 | | 0.453 | |  |
|  | Sports <2 hrs/wk vs. no sports | 1.58 | 0.56 | 2.60 | 0.003 |  | 2.87 | 1.22 | 4.52 | 0.001 |  | 1.60 | 0.72 | 2.48 | <0.001 |  | 2.96 | 1.41 | 4.51 | | <0.001 | |  |
|  | Sports ≥2 hrs/wk vs. no sports | 1.98 | 0.81 | 3.15 | 0.001 |  | 4.82 | 3.10 | 6.54 | <0.001 |  | 3.31 | 2.06 | 4.57 | <0.001 |  | 4.43 | 2.71 | 6.15 | | <0.001 | |  |
|  | Moderate drinking vs. no alcohol | 4.06 | 2.20 | 5.92 | <0.001 |  | 3.79 | 0.47 | 7.12 | 0.026 |  | 2.45 | 0.83 | 4.07 | 0.003 |  | 1.02 | -0.62 | 2.65 | | 0.222 | |  |
|  | Risky drinking vs. no alcohol | 4.05 | 1.99 | 6.10 | <0.001 |  | 3.11 | -0.50 | 6.73 | 0.091 |  | 2.80 | 0.88 | 4.72 | 0.005 |  | 1.11 | -1.21 | 3.43 | | 0.348 | |  |
|  | Multimorbidity (no vs. yes) | 3.51 | 2.55 | 4.47 | <0.001 |  | 4.56 | 2.91 | 6.22 | <0.001 |  | 2.84 | 1.81 | 3.87 | <0.001 |  | 4.67 | 3.27 | 6.06 | | <0.001 | |  |
|  |  |  |  |  |  |  |  |  |  |  |  |  |  |  |  |  |  |  |  | |  | |  |
| **Model 9s** | **DEGS1 vs. GNHIES98** | **1.39** | **0.55** | **2.23** | **0.001** | **0.150** | **0.46** | **-0.88** | **1.79** | **0.501** | **0.149** | **0.23** | **-0.72** | **1.17** | **0.639** | **0.180** | **0.35** | **-1.04** | **1.74** | | **0.617** | | **0.181** |
|  | Living with others vs. living alone | 0.70 | -1.07 | 2.46 | 0.437 |  | 0.47 | -1.49 | 2.42 | 0.637 |  | 0.61 | -0.67 | 1.88 | 0.348 |  | 0.44 | -1.06 | 1.95 | | 0.561 | |  |
|  | Middle vs. primary education level | 1.93 | 0.93 | 2.92 | <0.001 |  | 0.00 | -1.64 | 1.63 | 0.996 |  | 1.75 | 0.70 | 2.81 | 0.001 |  | 1.48 | 0.07 | 2.89 | | 0.040 | |  |
|  | High vs. primary education level | 2.39 | 1.40 | 3.39 | <0.001 |  | 1.27 | -0.21 | 2.76 | 0.091 |  | 0.88 | -0.48 | 2.24 | 0.204 |  | 3.64 | 1.66 | 5.62 | | <0.001 | |  |
|  | BMI<25 vs. ≥30 | 1.64 | 0.37 | 2.91 | 0.012 |  | 2.84 | 0.62 | 5.06 | 0.012 |  | 3.94 | 2.65 | 5.23 | <0.001 |  | 3.75 | 1.97 | 5.52 | | <0.001 | |  |
|  | BMI 25-30 vs. ≥30 | 1.67 | 0.66 | 2.67 | 0.001 |  | 2.12 | 0.39 | 3.85 | 0.016 |  | 2.65 | 1.35 | 3.96 | <0.001 |  | 2.41 | 0.96 | 3.86 | | 0.001 | |  |
|  | Ex-smoker vs. smoker | 0.73 | -0.45 | 1.91 | 0.222 |  | 0.21 | -1.82 | 2.24 | 0.838 |  | -0.40 | -1.77 | 0.98 | 0.571 |  | -0.61 | -3.19 | 1.97 | | 0.640 | |  |
|  | Non-smoker vs. smoker | 1.60 | 0.44 | 2.77 | 0.007 |  | 1.58 | -0.48 | 3.65 | 0.133 |  | -0.15 | -1.26 | 0.97 | 0.794 |  | 0.42 | -2.19 | 3.03 | | 0.752 | |  |
|  | Sports <2 hrs/wk vs. no sports | 1.15 | 0.12 | 2.17 | 0.028 |  | 2.71 | 1.26 | 4.17 | <0.001 |  | 1.74 | 0.85 | 2.63 | <0.001 |  | 2.95 | 1.54 | 4.36 | | <0.001 | |  |
|  | Sports ≥ 2 hrs/wk vs. no sports | 1.92 | 0.79 | 3.06 | 0.001 |  | 3.95 | 2.33 | 5.58 | <0.001 |  | 3.45 | 2.23 | 4.66 | <0.001 |  | 4.24 | 2.49 | 5.99 | | <0.001 | |  |
|  | Moderate drinking vs. no alcohol | 3.99 | 2.06 | 5.93 | <0.001 |  | 4.19 | 1.13 | 7.24 | 0.007 |  | 2.59 | 1.07 | 4.11 | 0.001 |  | 0.69 | -0.85 | 2.23 | | 0.379 | |  |
|  | Risky drinking vs. no alcohol | 3.67 | 1.58 | 5.77 | 0.001 |  | 3.08 | -0.25 | 6.42 | 0.070 |  | 2.88 | 1.03 | 4.73 | 0.002 |  | 1.29 | -0.92 | 3.49 | | 0.251 | |  |
|  | Polypharmacy (no vs. yes) | 6.53 | 4.65 | 8.40 | <0.001 |  | 5.60 | 3.95 | 7.26 | <0.001 |  | 6.24 | 4.64 | 7.83 | <0.001 |  | 6.74 | 5.39 | 8.09 | | <0.001 | |  |

Weighted and standardized to the population of 31.12.2010.

Change in mean: Derived from SPSS Complex Samples General Linear Model (CSGLM) with physical functioning as the dependent variable.

**Model 0**= survey (DEGS1 vs. GNHIES 98)

**Model 1**=Model 0+ living alone (yes vs. no). **Model 2**=Model 0+ education (low, medium, high). **Model 3**=Model 0+ BMI (<25, 25-30, ≥30 mg/m2). **Model 4**=Model 0+ smoking status (current, former and never). **Model 5**=Model 0+ regular sports activity (0, <2, ≥2 hour/week). **Model 6**= Model 0+ alcohol consumption (no, moderate and risky drinking). **Model 7**= Model 0+multimorbidity (yes, no). **Model 8**=Model 0+ polypharmacy (yes, no).

**Model 9**= survey (DEGS1 vs. GNHIES 98) + living alone (yes vs. no) + education (low, medium, high) + BMI (<25, 25-30, ≥30 mg/m2) + smoking status (current, former and never) + regular sports activity (0, <2, ≥2 hour/week) + alcohol consumption (no, moderate and risky drinking) + multimorbidity (yes, no).

**Model 9s**= survey (DEGS1 vs. GNHIES 98) + living alone (yes vs. no) + education (low, medium, high) + BMI (<25, 25-30, ≥30 mg/m2) + smoking status (current, former and never) + regular sports activity (0, <2, ≥2 hour/week) + alcohol consumption (no, moderate and risky drinking) + polypharmacy (yes, no).
